# Evaluation of a microcolony growth monitoring method for the rapid determination of ethambutol resistance in *Mycobacterium tuberculosis*

**DOI:** 10.1186/1471-2334-14-380

**Published:** 2014-07-10

**Authors:** Alice L den Hertog, Sandra Menting, Ernst T Smienk, Jim Werngren, Sven Hoffner, Richard M Anthony

**Affiliations:** 1 Royal Tropical Institute, KIT Biomedical Research, Meibergdreef 39, 1105, AZ, Amsterdam, The Netherlands; 2 Department of Diagnostics and Vaccines, Unit of Highly Pathogenic Microorganisms, Swedish Institute for Communicable Disease Control, S-171 82, Solna, Sweden

**Keywords:** Ethambutol, Drug susceptibility testing, *Mycobacterium tuberculosis*, Culture

## Abstract

**Background:**

Due to the increasing prevalence of *Mycobacterium tuberculosis* strains resistant to one or more antibiotics, there is a need for new quantitative culture methods both for drug susceptibility testing and for validation of mutations putatively associated with drug resistance. We previously developed a (myco) bacterial culture method, in which multiple growing microcolonies are monitored individually. Transfer of the growing microcolonies to selective medium allows the effect on the growth rate of each individual colony to be determined. As entire growing colonies are exposed to antibiotics rather than re-subbed, a second lag phase is avoided and results are obtained more rapidly. Here we investigate the performance of the microcolony method to differentiate between ethambutol (EMB) resistant, intermediate and susceptible strains.

**Methods:**

One week old microcolonies from a reference panel of four strains with known EMB susceptibility were transferred to different concentrations of EMB. Growth rates during the 1^st^ 2 days of exposure were used to set up classification criteria to test and classify a blinded panel of 20 tuberculosis strains with different susceptibilities.

**Results:**

For 18 strains (90%) reference culture results corresponded to our classifications based on data collected within 9 days of inoculation. A single strain was classified as Intermediate instead of Susceptible, and 1 strain could not be classified due to a contamination.

**Conclusions:**

Using a microcolony growth monitoring method we were able to classify, within 9 days after inoculation, a panel of strains as EMB susceptible, intermediate or resistant with 90% correlation to the reference methods.

## Background

The increasing prevalence of *Mycobacterium tuberculosis* strains resistant to one or more antibiotics makes drug susceptibility testing (DST) a high priority. The rapid molecular detection of resistance e.g. for rifampicin (RIF) by automated molecular methods [1] creates an additional demand for phenotypic susceptibility testing to other agents. In fact a complete prediction of the *in vivo* susceptibility of strains to available drugs is desired both to ensure the provision of the least toxic and most effective therapy to the individual patient and for interrupting the transmission of drug resistant pathogens. For some drugs this is difficult to achieve with the standard, at most semi-quantitative culture methods currently in use.

Unfortunately DST for tuberculosis (TB) is too slow, complex or dangerous to be effectively implemented in all settings, therefore novel strategies with the potential to be automated warrant investigation [2]. We have previously described an in-house (myco) bacterial culture method [3], in which the growth of microcolonies from individual colony forming units (CFUs) is monitored similar to the ScanLag system [4]. But in addition, in our system the microcolonies can be transferred to different media whilst monitoring their growth. Thus, it is possible to determine the susceptibility of growing colonies to a drug by measuring the change in growth rate after exposure. As each individual colony is monitored, detection of multiple phenotypes, for example a mixed resistant and susceptible bacterial population (heteroresistance), is possible [3].

Detection of RIF susceptibility and resistance in *Mycobacterium tuberculosis* lab strains was previously achieved within 8 days after inoculation and only 24 hours after exposure to RIF [3]. Transferring intact growing colonies onto selective medium during their exponential growth phase avoids the second lag phase and thus is more rapid than classical subculturing selected colonies onto plates or into liquid medium containing antibiotics. Also, as individual microcolony growth rates can be accurately monitored, more subtle effects may be detected such as partial inhibition. This may aid the identification of clinically significant resistance in strains that (due to an MIC close to the breakpoint concentration) is more difficult to detect using traditional methods. Also the ability of the system to detect more subtle *in vitro* responses to antibiotic challenge may allow the detection of additional phenotypes with altered response to *in vivo* therapy. Therefore, we investigated the capacity of our microcolony approach for DST of a more challenging antibiotic Ethambutol (EMB).

Standard methods for DST are based on either the proportion method and performed on solid media, or on critical concentration assays performed in liquid culture systems [5]. More recently the observation of cording/“microcolony” formation in liquid media in the Microscopic Observation Drug Susceptibility assay (MODS) has been applied [6,7].

For some antimycobacterial drugs such as isoniazid and RIF, the agreement between different methods and between different labs is very good , but for EMB, even in well-established labs susceptibility testing remains challenging [8,9], possibly because the minimal inhibitory concentrations (MICs) of both wild type and EMB resistant strains are close to the recommended critical concentration [10]. Therefore maintaining reproducibility becomes more difficult than for antibiotics such as RIF, for which the majority of resistant strains have a MIC far above the breakpoint concentration [11,12].

Also, susceptibility testing against EMB does not correlate well with the known molecular markers of EMB resistance thus molecular testing produces results that are difficult to interpret. A number of mutations have been associated with EMB resistance, predominantly in embCAB [13,14], but their association with resistance is not strong enough to classify strains as susceptible or resistant [14].

Here we investigate the performance of the microcolony method to differentiate between EMB resistant, intermediate and susceptible strains. Using four strains with known EMB susceptibility/resistance we determined microcolony growth classification criteria. Then the growth of microcolonies from a blinded panel of 20 TB strains was monitored and these strains classified using our classification criteria. Results were then compared to classical susceptibility testing performed in a reference laboratory.

## Methods

### Strains

A panel of 24 TB strains was received by the Royal Tropical Institute from the Swedish Institute for Communicable Disease Control (SMI) on Lowenstein Jensen slopes (Table 1). Strains were coded 1–24 and from strains 1–4 the susceptibility testing results were provided to serve as reference. Strain 1 was labeled susceptible (S), strain 2 intermediate (I) and strains 3 and 4 resistant (R). No information was provided from strains 5–24 prior to communication of the results to the SMI.

**Table 1:** Classification of the tested strains

**strain**	**SMI Strain number**	**Threshold concentration (mg/L) †**	**Microcolony growth-based classification (This study)**	**SMI classification**
1	XTB 10-172	1	S	S
2	XTB 09-045	4	I	I
3	XTB 09-023	16	R	R
4	XTB 09-060	16	R	R
22#	XTB 09-060	>8	R	R
24#	XTB 09-060	>8	R	R
5	XTB 10-167	≤1	S	S
6	XTB 10-166	4	**I**	**S**
7	XTB 10-158	≤2	S	S
8	XTB 09-109	>8	R	R
9	XTB 09-108	8	I/R*	**I**
10	BTB 09-554	>8	R	R
11	BTB 11-250	4	I	I
12	BTB 11-214	>4	R	R
13	BTB 11-145	2	S	S
14	BTB 11-421	2	S	S
15	XTB 10-170	>8	R	R
23#	XTB 10-170	>4	R	R
16	BTB 11-416	4	I	S/I
17	BTB 11-417	≤1	S	S
18	BTB 11-418	≤1	S	S
19	BTB 11-419	≤1	S	S
20	BTB 11-420	≤1	S	S
21	BTB09-553	>4	R	R

Strains 1–4: reference strains, strains 5–24: blinded strains. † Threshold concentration, lowest concentration tested at which >40% reduction in growth rate compared to control (no EMB) was recorded. Where 40% reduction in growth rate was reached at the lowest concentration tested a ≤ symbol precedes the concentration, Where the threshold was not reached at the highest concentration tested, this is indicated by a > symbol. *4 mg/L missing due to contamination. When retested, strain 9 was correctly classified as intermediate. # Strains 22 and 24, and strain 23 were (unknown) duplicates of strain 4 and 15, respectively. The 2 results discordant between the microcolony growth-based classification and the SMI classifications are indicated in boldface.

All strains were inoculated in Middlebrook (MB) 7H9 medium (Difco, BD, Sparks, MD, USA) + OADC (BBL, BD) and subcultured every 3–4 weeks.

To control laboratory procedures multiplex ligation-dependent probe amplification (MLPA) [15] was performed on DNA isolated from the 1^st^ routine cultures and from the last subcultures after testing of the panel, this yielded identical results for all (45) resistance associated markers and lineage type markers for all individual strains (data not shown).

### DST performed with BACTEC MGIT 960 and Middlebrook proportion method

Strains were tested to 4 and 8 mg/L EMB (Sigma-Aldrich, Stockholm, Sweden) using the Middlebrook 7H10 agar proportion method as earlier described [10] or to 2.5 and 5 mg/L using the BACTEC MGIT 960 system (Becton Dickinson, Sparks, MD).

For the BACTEC MGIT, the DST inoculum was prepared from bacterial growth on Löwenstein-Jensen egg medium in 37°C. Briefly, two 1 μL-loops of bacteria were suspended in 3 ml of phosphate buffered saline (PBS) in a small glass tube with glass beads. Homogenization of the bacterial suspension was obtained by vortexing or in an ultrasound water bath to disperse clumps. Thereafter the suspension was left to sediment for 20 min and the upper 2 mL were transferred to a new tube and let to sediment for another 15 min.

Prior to inoculation of the BACTEC MGIT culture tubes the bacterial suspension was adjusted to a McFarland turbidity of 0.5 and diluted in PBS according to the test protocol from the manufacturer. The drug susceptibility to the recommended critical concentration of 5 mg/L EMB was assessed using the Becton Dickinson kit.

Strains were determined EMB susceptible when the growth unit (GU) of the culture tubes containing 2.5-5 mg/L EMB were ≤100 when the 1:100 diluted drug free control had reached GU = 400. Strains growing at concentrations between 2.5 and 5 mg/L were considered intermediary (I) resistant.

### Microcolony growth monitoring

Microcolony growth was performed in 4-well culture plates (Nunc multidish 4 SI, Nunc A/S, Denmark), on squares of porous aluminium oxide (PAO, Whatman, Kent, UK) on top of MB7H10 agar (Difco, BD, Sparks, MD, USA) + OADC. Agar was prepared in standard petri dishes, and disks punched out of the agar were transferred to the 4-well culture plates. Strips of 3.6×0.8 cm PAO were sterilized by submerging in 100% ethanol and cut into 4 squares of approx. 0.8×0.8 cm. After evaporation of the ethanol PAO squares were wetted in sterile water and placed on MB7H10 + OADC agar in the 4-well culture plates.

Mycobacterial cultures were vortexed for 20s and sedimented for at least 15 min. A 2 ml aliquot of the suspension was filtered through a 5 μm filter (Whatman) to obtain a suspension of single cells. Cells were diluted in MB7H9 to approx. 3× 10^6^ cells/ml based on the assumption that an OD_450_ of 0.15 corresponds to 10^8^ cells/ml. Three μl of this suspension was inoculated on the squares of PAO. Plates were incubated at 36°C.

A stock solution of 4 mg/ml EMB in water was prepared and stored aliquoted at −20°C. Four-well plates with MB7H10 agar + OADC supplemented with different concentrations of EMB (Ethambutol dihydrochloride, Sigma-Aldrich, St. Louis, MO, USA) were prepared as described above and used within 9 days.

**Figure 1:**
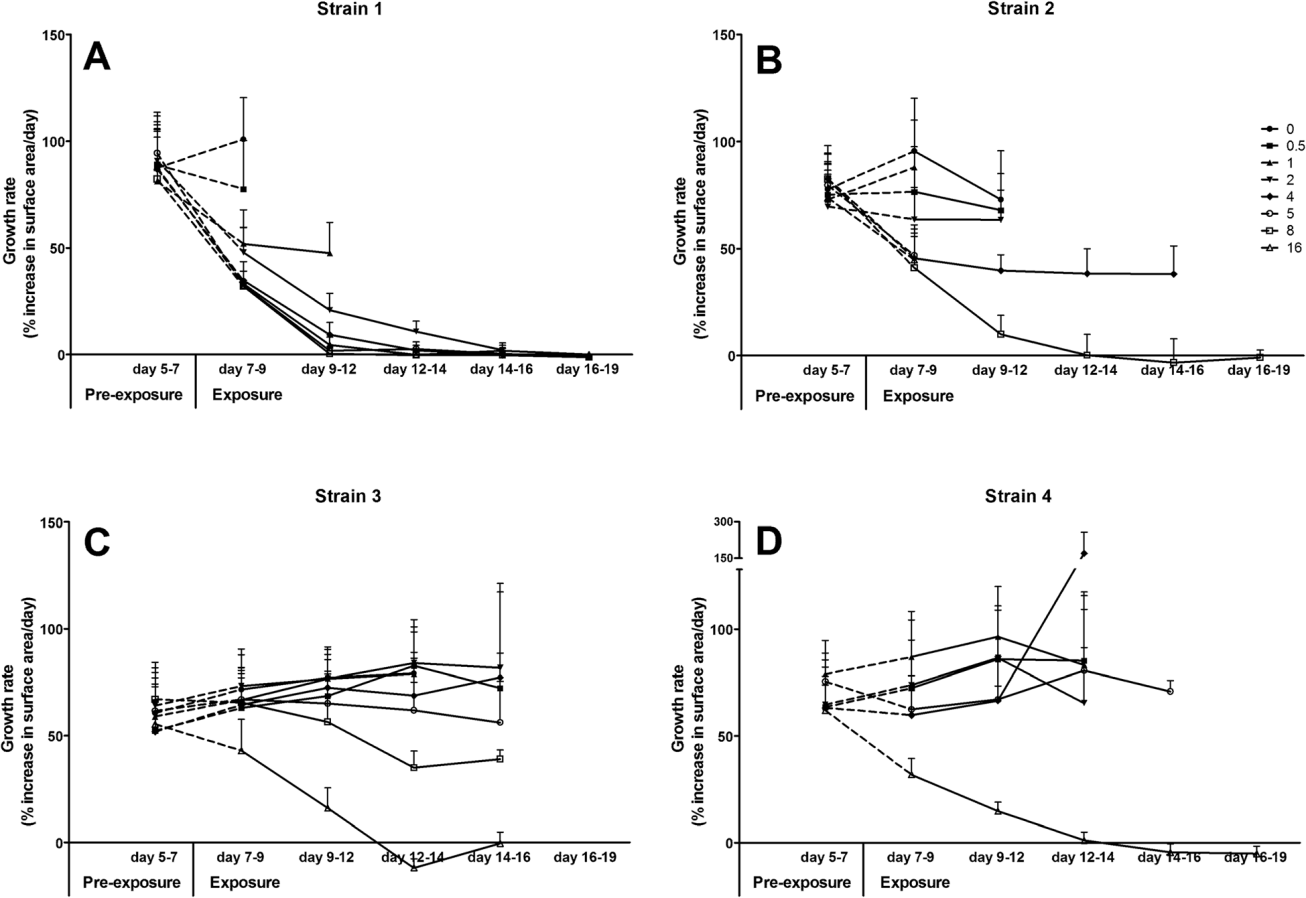
**Growth rates of the reference strains 1 (A), 2 (B), 3 (C) and 4 (D) before and after transfer to EMB.** Note that the control of strain 1 was fully grown at day 12, thus control growth rate could not be calculated beyond the 2^nd^ day of exposure. Average and standard deviations are shown. Data from strain 2 exposed to 1, 5 and 16 mg/L EMB and from strain 4 exposed to 0 and 8 mg/L EMB are missing due to contamination.

**Figure 2:**
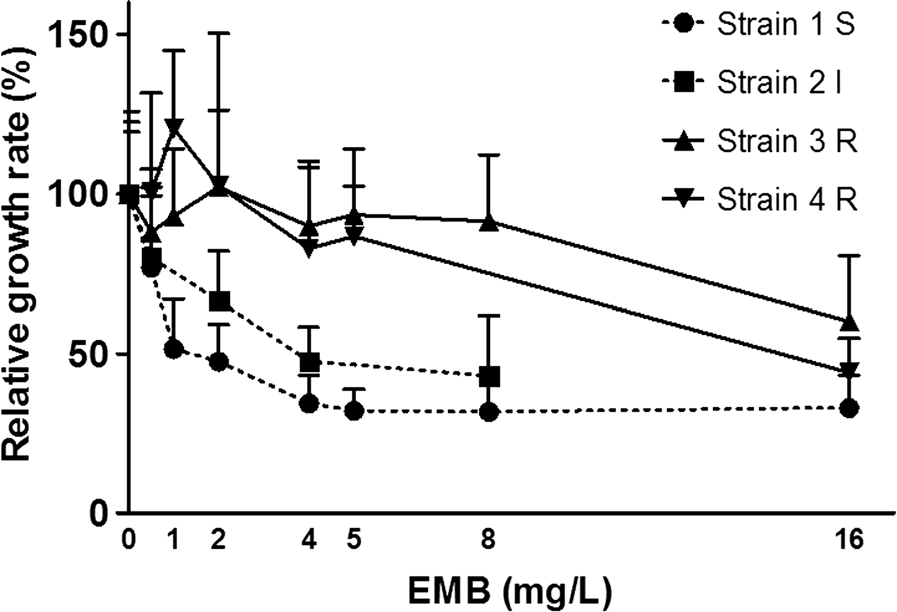
**Relative growth rates of reference strains (as % of 0 mg/L control) between days 7 and 9 after transfer to EMB on day 7.** *For strain 4 results are shown relative to the pre-exposure growth rate due to contamination of the control. Error bars represent the standard deviation of the relative growth rate of individual microcolonies.

Initially, growth of all 24 strains on non selective medium was monitored between days 5 and 16 or until the colonies covered the surface to determine the time frame for the EMB exposure experiments. Based on the results of experiments, exposure experiments to EMB were set up as follows:

For the four reference strains 1–4 eight squares of PAO were inoculated and grown for 7 days on non-selective medium. The PAO squares containing microcolonies were manually transferred to 0; 0.5; 1; 2; 4; 5; 8 and 16 μg/ml EMB. Using an automated microscope system (Muscan, CCM BV, Nuenen, The Netherlands), images were recorded at 5 and 7 days after inoculation (pre-exposure baseline), directly after transfer (exposure to EMB began 7 days after inoculation) and then after 2, 5, 7 and 9 days of EMB exposure (corresponding with days 7, 9, 12, 14 and 16 after inoculation). Imaging was terminated at 16 days or if single colonies could no longer be discriminated due to overgrowth.Based on the results of the four reference strains 1–4 (Figures 1 and 2) it was decided to test the blinded panel using the same conditions and transfer on day 7, except that exposures were limited to concentrations of 0; 1; 2; 4 and 8 μg/ml EMB. Imaging was performed at the same time points as above.

#### Image and data analysis

Image analysis was performed essentially as described in den Hertog et al. [3], but using a second generation microscope system (Muscan) allowing for higher throughput, and another software package with additional tools (FIJI; [16]).

Microcolony microscopy was performed using an automated microscopy system (Muscan) with bright field imaging, coaxial illumination, and a 5 × objective. Images were recorded with a monochrome CCD camera (XCD-SX90, Sony, Tokio, Japan; with field size 1280×960 pixels). From each well at each time point a set of 81 images (9 by 9 images of 0.95 × 0.71 mm each with an overlap of approx. 5.6% and 16.2% in X and Y direction respectively) was collected covering a total area of approx. 8.0 × 5.5 mm.

Using FIJI, first the set of 81 photos recorded per PAO square were stitched into a single image using the “Stitching 2D/3D” plugin [17]. Next, all stitched TIF images of the same PAO taken at the different time points were stacked and registered using the “Linear stack alignment with SIFT” plugin [18] to align potential colonies over all time points. Standard settings were used except the minimum and maximum image size for the scale invariant Interest point detector were set at 200 and 3000 respectively.

Macros were written to perform the following steps in FIJI: first a background reduction (with rolling ball radius of 200 pixels) and a 2 pixel radius median filter were applied. Next, using the “RenyiEntropy” method the images were converted to binary with a threshold level of 238. Using the “Analyse particles” tool the sizes and XY coordinates of all particles with a minimal size threshold of 50 pixels and a circularity of or above 0.5 were saved into a text file.

The data extracted from the images was then processed using the web application described by den Hertog *et al.*[3] to identify particles with equivalent XY coordinates present in at least two sequential time points. The sizes of the resulting lists of objects at sequential time points were exported to excel for further analysis. On basis of this information individual colony growth rates, defined as % increase in surface area/day, were calculated for all strains and conditions between sequential time points.

Objects that fulfilled the following were identified as mycobacterial colonies:

An increase in surface area of the object of >20% per day between days 5 and 7 after inoculation.

No significant effect of transfer on the objects surface area (<20% change); i.e. similar surface area of both measurements on day 7 after inoculation made before and after transfer

Presence and no decrease in size (<10% per day) of the object after transfer to selective medium (or control) days 7 and 9.

#### Classification

Based on the growth inhibition profiles of the reference strains 1–4 (See Results section), criteria were defined to allow the unknown strains to be classified. At each concentration of EMB the relative average growth rate compared to the unexposed control (0 mg/L EMB) was calculated for days 7–9, 0 to 2 days EMB exposure. Strains were classified based on the lowest EMB concentration resulting in a decrease in relative growth rate (compared to the unexposed control) of > 40%; If growth was decreased 40% or more at i. concentrations ≤2 mg/L, strains were classified as Susceptible, ii. 4 mg/L as Intermediate, iii. if growth rate was reduced less than 40% at >4 mg/L as Resistant.

## Results

### Baseline growth rate of strains on non selective medium

**Figure 3:**
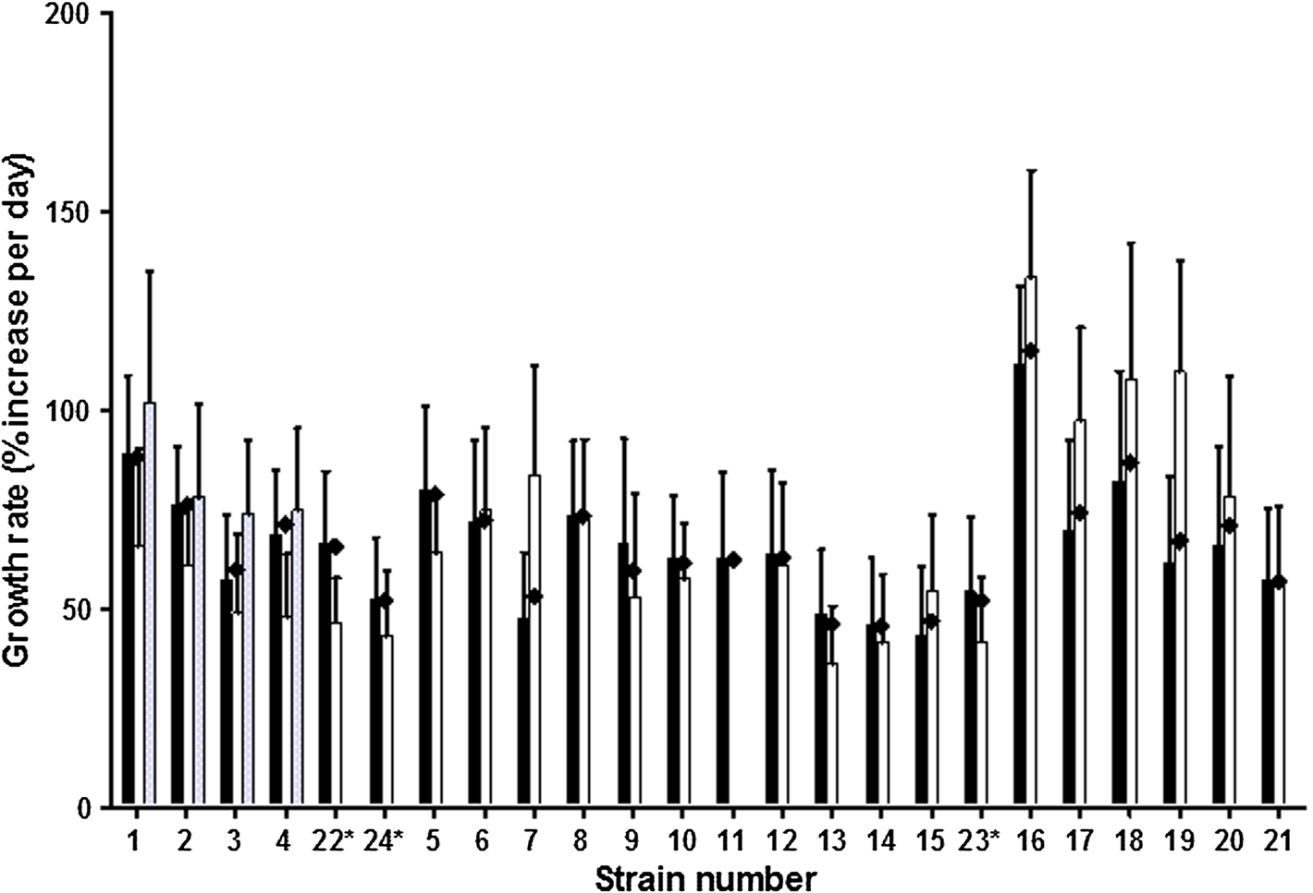
**Growth rates (% increase in surface area per day) of strains 1–24 between days 5 and 7 after inoculation.** Pre-exposure data from the EMB exposure experiments (black bars), and data collected in the baseline experiments (white and grey bars) are combined. Diamonds represent the weighted average of all data points for each strain, ranging between 629 and 9608 (median 3911.5) data points per strain. *As shown in Table 1, strains 22 and 24 are the same strain as strain 4, and strain 23 is the same as strain 15, although they were included as individual strains in the blinded panel. Error bars represent the standard deviation of the growth rate of individual microcolonies.

Growth of all strains was monitored from 5 days to 16 days after inoculation or until confluence was reached. The data collected was used to determine for the EMB exposure experiments at which time points to measure the baseline and transfer the microcolonies to EMB. All strains had stable growth rates measured between days 5–9 and when data was available, between days 5–12. All strains were thus in the exponential growth phase between days 5 and 9. Based on data from 629 to 9608 (median 3911.5) microcolonies per strain the average microcolony surface area between days 5 and 7 after inoculation increased by 45.6% to 114.7% per day (Figure 3). These growth rates correspond to doubling times of 20.9 to 52.6 hours.

Despite the wide range in growth rates, microcolonies from all strains were detectable 5 days after inoculation. Therefore, imaging was begun at day 5 and exposure to EMB at day 7.

### Effects of EMB on the growth of reference strains

To establish criteria to classify the blinded strains, four reference strains with known EMB susceptibility (susceptible, intermediate and resistant) were exposed to 0–16 mg/L EMB from day 7 after inoculation and growth curves for individual microcolonies were produced (Figure 1).

The growth rate of the susceptible strain 1 was approximately halved by the presence of 1 mg/L EMB within the 1^st^ 2 days of exposure (days 7–9). After day 9 (2 days EMB exposure) the growth of strain 1 was completely inhibited by concentrations higher than 4 mg/L (Figure 1A). In contrast, the growth rate of intermediate resistant strain 2 was unaffected by the presence of up to 2 mg/L EMB for the 1^st^ 5 days of exposure (day 12) but clear inhibition was seen for concentrations over 4 mg/L EMB from day 7 onwards (Figure 1A). The growth rate of resistant strains 3 and 4 was only reduced by 16 mg/L of EMB when compared to the antibiotic free control or the pre-exposure growth rate (Figure 1C-D).The data collected between days 7 and 9 presented in Figure 1 was used to calculate the relative growth rates compared to the unexposed controls (0 mg/L for all EMB concentrations) for each strain (Figure 2). Based on this analysis it was decided to expose the strains in the blinded panel to 0; 1; 2; 4 and 8 mg/L EMB. Furthermore a classification scheme was established as follows: Strains with a decrease in growth rate compared to the unexposed control between 0–2 days of exposure of more than 40% at <2 mg/L were classified as susceptible, a decrease in growth rate of >40% between 2–4 mg/L as intermediate, and a decrease in growth rate of <40% at 4 mg/L as resistant. The resulting classification was then reported to the reference laboratory and only then the code broken.

**Figure 4:**
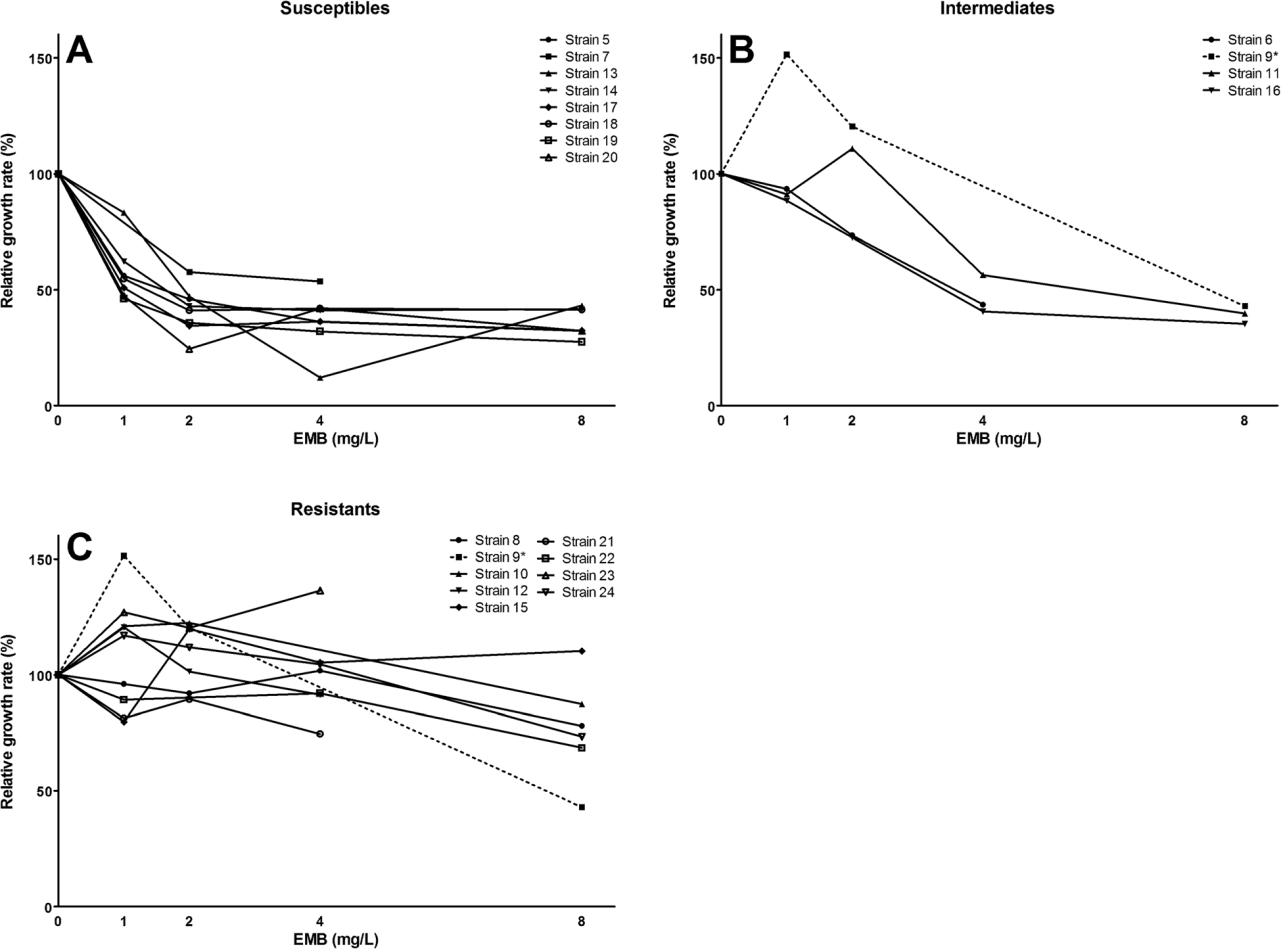
**Classification of the blinded panel of strains as susceptible (A), intermediate (B) or resistant (C) based on their relative growth rates on EMB containing medium between day 7–9.** *Strain 9 could not be classified between I and R due to missing data due to contamination of the sample exposed to 4 mg/L EMB and is shown in both I and R graphs. The 0 mg/L control of strain 19 was contaminated, therefore pre-exposure growth rate was used as 100% for this strain. When retested (not shown) results for strain 19 were similar.

Using the microcolony method eight strains were classified as susceptible (Figure 4A, Table 1), three strains as intermediate (Figure 4B) and eight strains as resistant (Figure 4C). Classification of strain 9 was uncertain (between intermediate and resistant Figure 4B-C) due to missing data, contamination of the 4 mg/L PAO filter. Upon breaking the code and comparing to the classification and MICs of the strains as determined by the Swedish Institute for Communicable Disease Control (Table 1) all resistant strains (strains 8, 10, 12, 15, 21, 22, 23 and 24) were found to have been correctly identified. Classification of the remaining strains all with a MICs below the breakpoint concentration of 5 mg/L was in agreement (intermediate or susceptible) by both methods except for strain 6 which was identified as intermediate by the microcolony method and susceptible by the reference laboratory (Table 1) and strain 9 which was not grouped due to missing data. Retesting of strain 9 after breaking the code resulted in corresponding classification as intermediate (data not shown).

Frequency distributions of the relative growth rates between days 7–9 of the exposed microcolonies showed homogeneous responses in all conditions for every strain (Additional file 1).

## Discussion

EMB susceptibility testing of *M. tuberculosis* isolates is challenging and difficult to perform reproducibly [19]. Here we have shown that with our microcolony growth monitoring method exposure of growing microcolonies to EMB can provide clear and accurate EMB susceptibility determinations within 2 days of exposure (9 days after initial inoculation). In this study the growth of a total of more than 100,000 automatically identified microcolonies was tracked.A blinded panel of 20 strains was tested, 18 of which were classified by our method into identical groups as the reference laboratory using the gold standard method. One strain (strain 6) classified by our method as intermediate was classified as susceptible by the reference laboratory. Another strain (strain 9) could not be classified more accurately than “Intermediate or Resistant” from the initial blinded testing due to laboratory contamination of the 4 mg/L exposure (Figure 4) , although when retested after breaking the code it was correctly classified as Intermediate by the microcolony method (not shown).

We classified the strains on the basis of % growth inhibition compared to an antibiotic free control at 2 concentrations (2 and 4 mg/L of EMB). However our method results in an unprecedented amount of data and it may be possible, if growth conditions are carefully selected, to determine EMB susceptibility by testing at single time point with only a single concentration. For example, after breaking the code we realized that the relative growth rate after 48 hours on 2 mg/L alone would have been sufficient to classify 17 out of 20 strains identically to the reference method (by classifying strains as sensitive strains with a relative growth rate <60%, strains as intermediate with a growth rate between 60 and 80%, and strains as resistant with a growth rate of >80% of the control (Figure 4)). Also in this study we decided to classify after 2 days of EMB exposure but findings of de Steenwinkel *et al.*[20] demonstrate that EMB inhibition in liquid cultures is detectable within 24 Hours. As our method can also detect subtle changes in growth we believe classification may have been possible after only 1 days exposure to EMB.

A number of mutations, predominantly in the *embB* gene, associated with EMB resistance have been identified, but the correlation with susceptibility testing is low [21,22] making the clinical significance of the mutations based on classical phenotypic testing results difficult to determine [23]. In a recent study [24], the spectrum of EMB resistance associated mutations has been expanded with other genes involved in decaprenylphosphoryl-β-D-arabinose (DPA) biosynthetic and utilization pathways both *in vitro* and *in vivo*. The authors demonstrate that resistance is acquired or increased step-wise, and multiple mutations in different genes including *embCAB* are required to obtain high level resistance. The broad spectrum of mutations that diversely affect the susceptibility explains the difficulty of phenotypic testing for EMB when compared to some other antibiotics for which the number of (identified) relevant resistance conferring mutations are limited and their effect on the MIC is dramatic. This results in poorer agreement between DST from different labs for EMB compared to other antibiotics such as RIF and isoniazid [10,19].

We have shown that inhibition of growth is not always complete, which would be an additional complication for traditional DST assays that can yield a positive or negative result at each concentration. For instance, the intermediately susceptible strain 2 continues to grow for at least 10 days with a constantly reduced growth rate of approx. 50% on 4 mg/L EMB (Figure 1). This partial inhibition was confirmed to be due to reduced growth rate of all colonies and thus is not an artifact due to heteroresistance (eg. a mixed genotype), which would have been detected as a proportion of colonies growing at the normal rate and a proportion completely inhibited (not shown, except for days 7–9 in Additional file 1).

The large numbers of mutations involved in EMB resistance make the development of accurate molecular assays for detecting resistance associated mutations as proxy for phenotypic susceptibility a huge challenge. In order to model the subtle effects of the different mutations on susceptibility to EMB, accurate measurement of the inhibition of growth rate of a statistically robust number of colonies in the presence of EMB may be more informative than only the MIC value resulting from traditional culture methods.

For this study, our data analysis treated all colonies from a single exposure as a homogeneous population, which was confirmed to be true based on the frequency distributions of growth rates between days 7–9 (Additional file 1). However, as the number of colonies analyzed per condition is large (generally >100), and analysis based on the frequency distributions of growth rates would be a powerful tool for detecting heteroresistance or confirming a single phenotype as was the case in this study.

Presently our method requires the careful manipulation of the PAO filters containing growing microcolonies and a simple device to facilitate this step will be required before the method can be widely adopted. Current we and our partners are working on the development of such a device as well as integrating our image analysis method into a user friendly microscopic reader with integrated software in order to make a system available to other researchers and more suitable to high throughput applications.

## Conclusion

In conclusion, monitoring of multiple microcolonies allows rapid determination of EMB susceptibility and has applications in diagnostics and for characterization of the inhibitory effect of EMB on strains carrying resistance associated mutations.

## Abbreviations

CFU: Colony forming unit; DST: Drug susceptibility testing; EMB: Ethambutol; GU: Growth unit; I: Intermediate; MIC: Minimal Inhibitory concentration; MLPA: Multiplex ligation-dependent probe amplification; PAO: Porous aluminum oxide; PBS: Phosphate Buffered saline; R: Resistant; RIF: Rifampicin; S: Susceptible; TB: Tuberculosis.

## Competing interests

The Royal Tropical Institute has a financial stake in the culture system and has a patent describing the culture system.

## Authors’ contributions

AH and RA designed the study, defined classification criteria and drafted the manuscript. SM and ES carried out the experiments. ES and AH performed the data analysis. JW and SH set up the strain panel, provided the reference results and helped draft the manuscript. All authors read and approved the final manuscript.

## Pre-publication history

The pre-publication history for this paper can be accessed here:

http://www.biomedcentral.com/1471-2334/14/380/prepub

## Supplementary Material

Additional file 1 **Frequency distributions of relative growth rates of strains 1–24 between days 7–9.** Frequency distributions of the growth rate relative to the averaged unexposed 0 mg/L control are shown for each strain. Distributions are shown with 20% bins, and data are plotted at the upper limit of each bin. (e.g. data over 80-100% is plotted at 100%). Frequency is expressed as the % of total counts. Click here for file
