# Supplementary material for: Evaluation of a microcolony growth monitoring method for the rapid determination of ethambutol resistance in Mycobacterium tuberculosis
Source: BMC Infect Dis. 2014 Jul 10;14:380. doi: 10.1186/1471-2334-14-380 (PMC4227065; doi:10.1186/1471-2334-14-380)

Additional File 1.docx


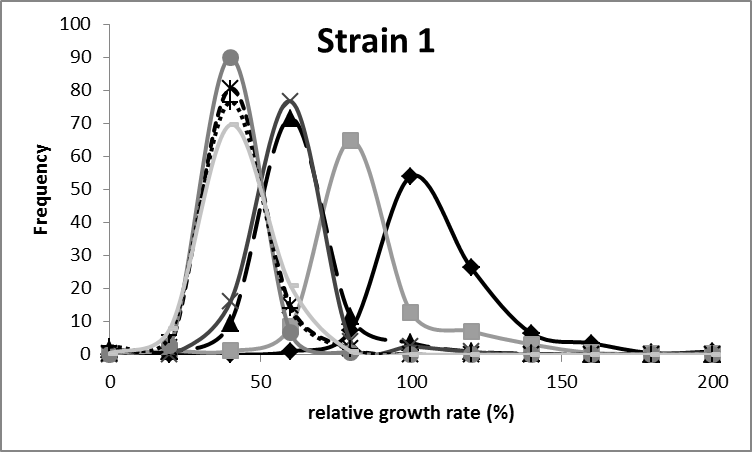


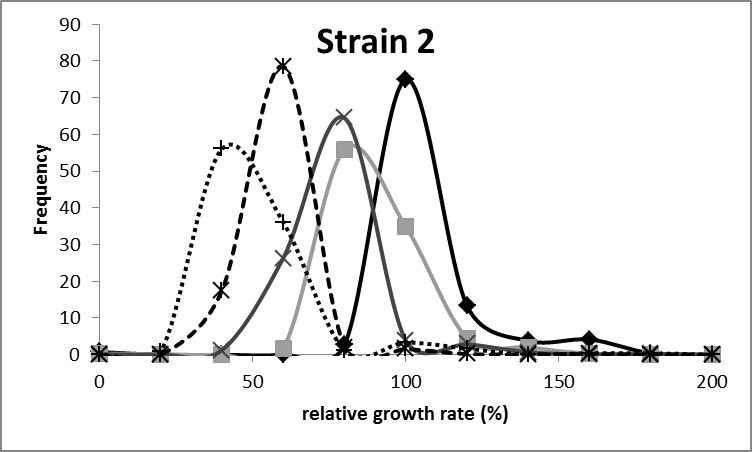


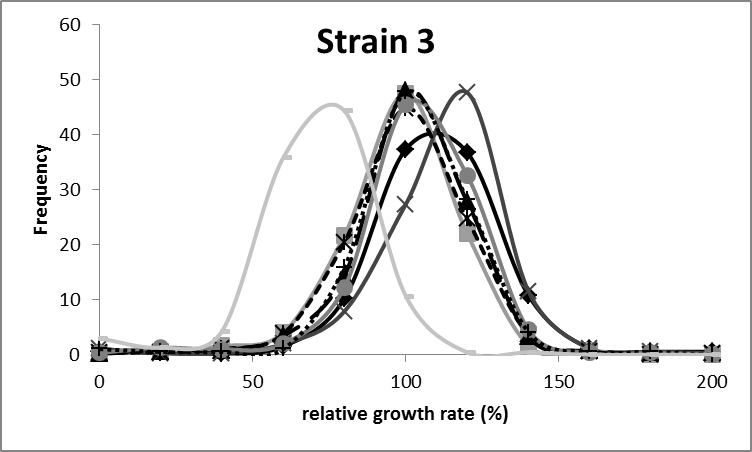


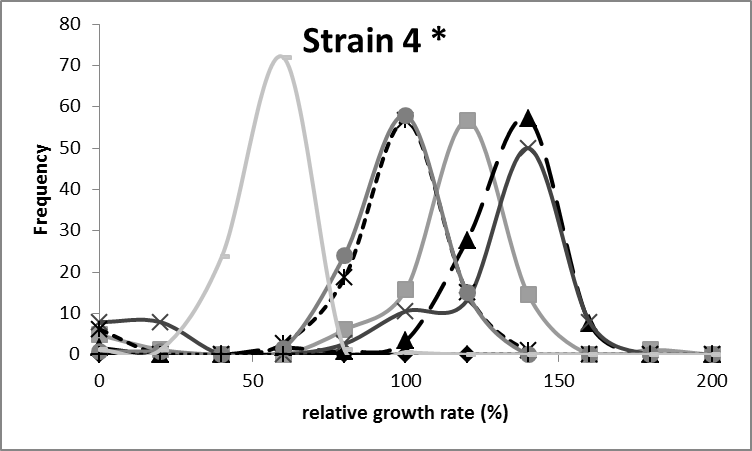


* Relative growth rate as compared to 0.5 mg/L due to contamination in 0 mg/L control


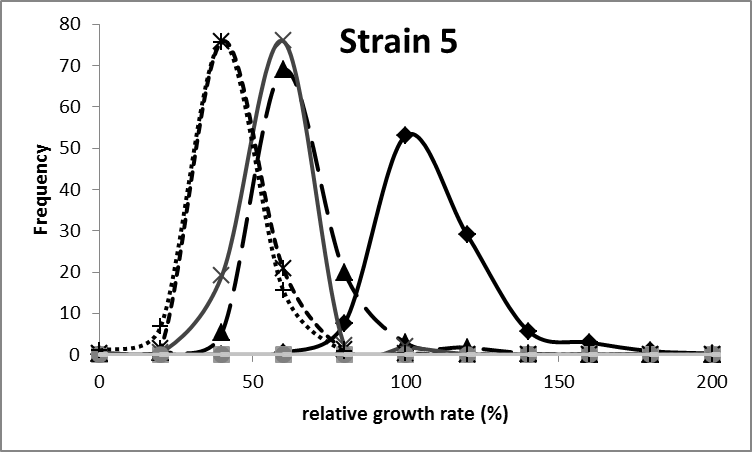


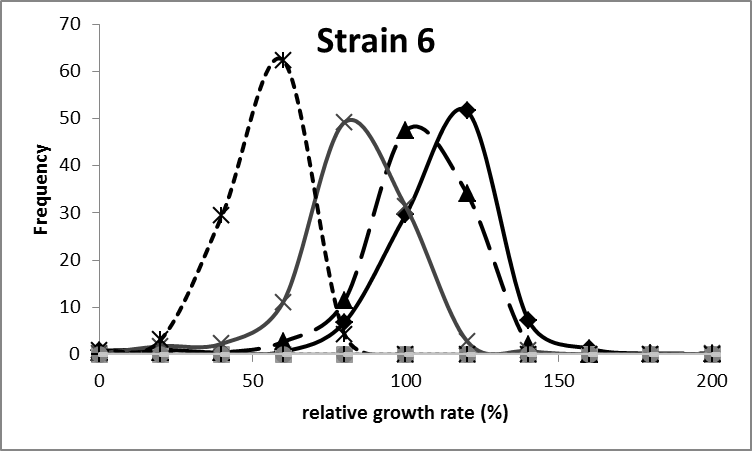


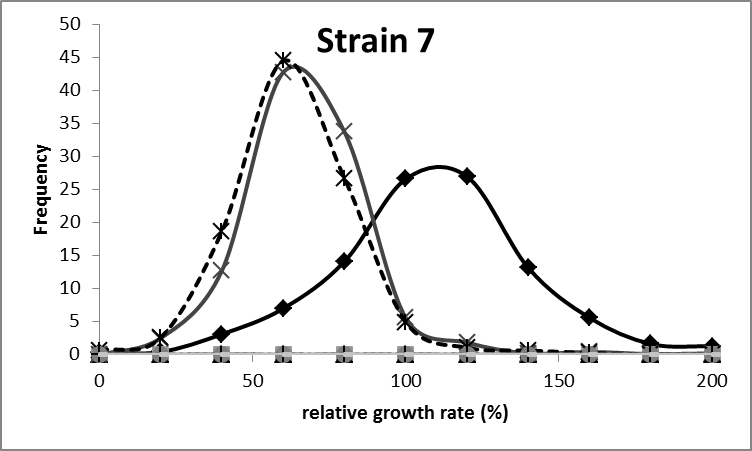


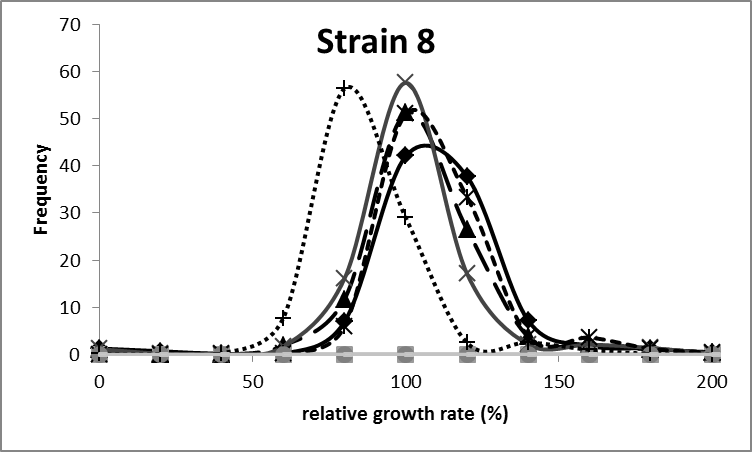


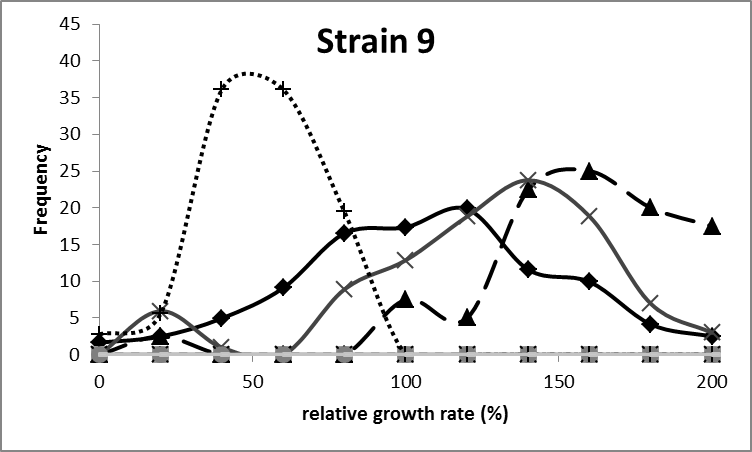


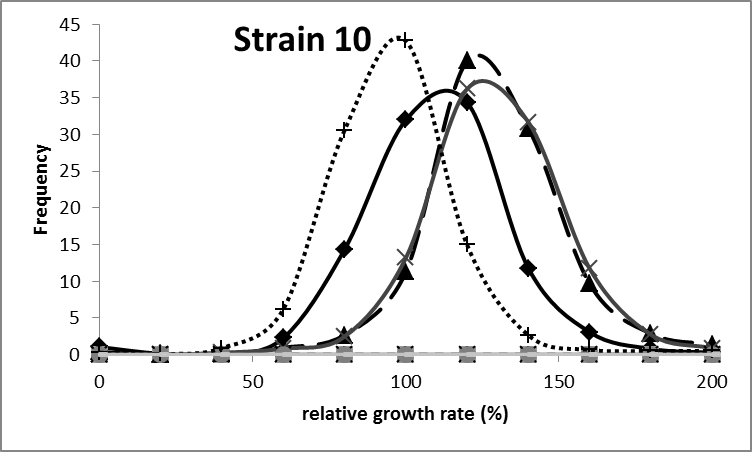


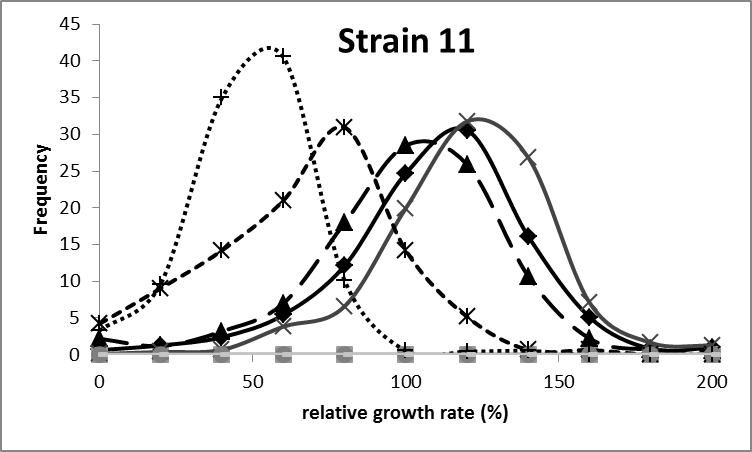


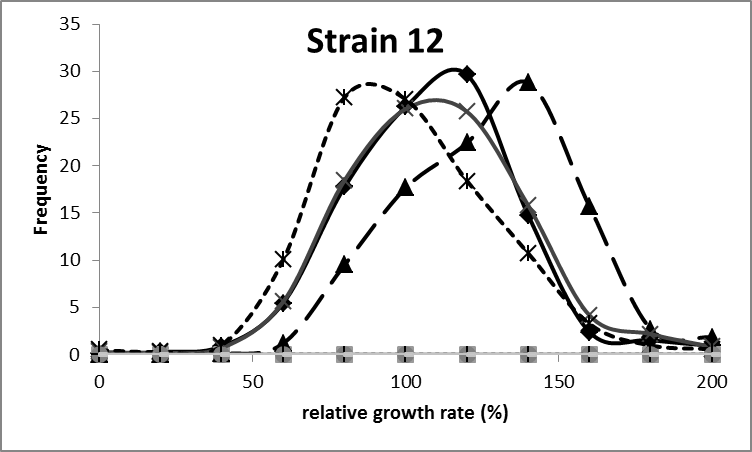


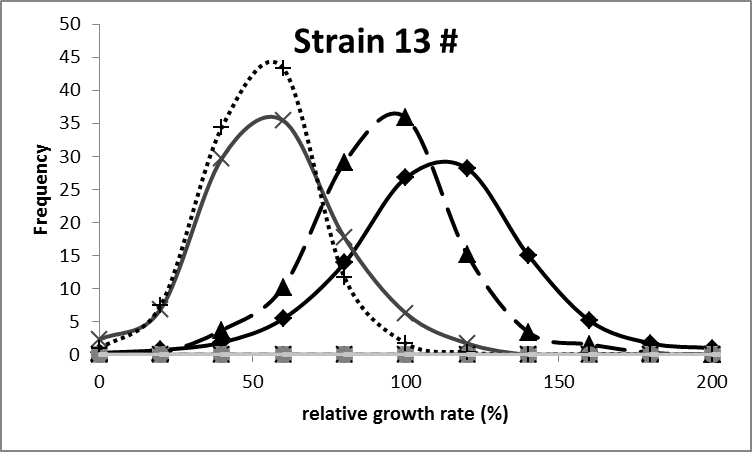


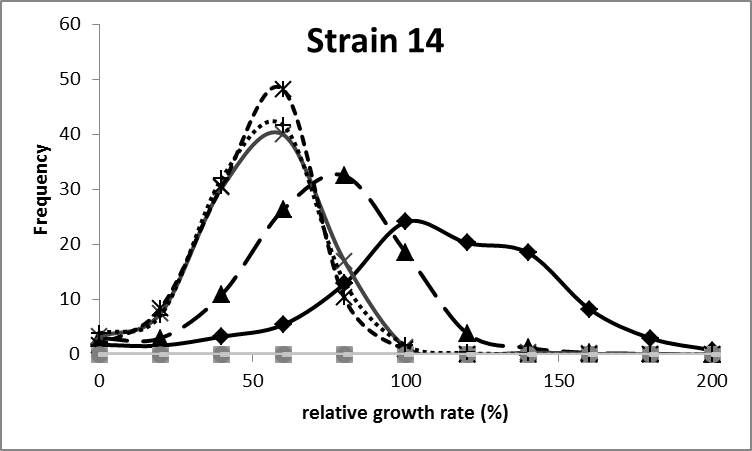


# Curve of 4mg/L not shown due to low counts


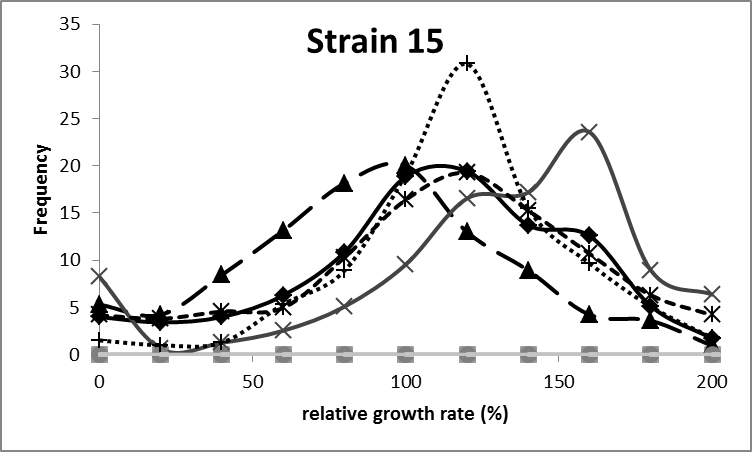


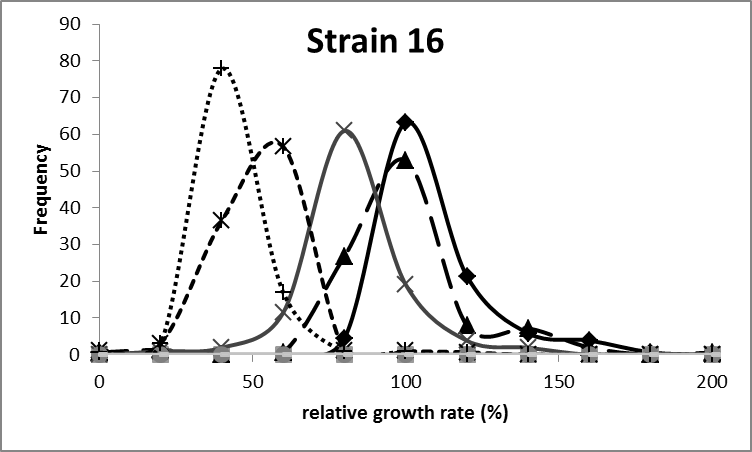


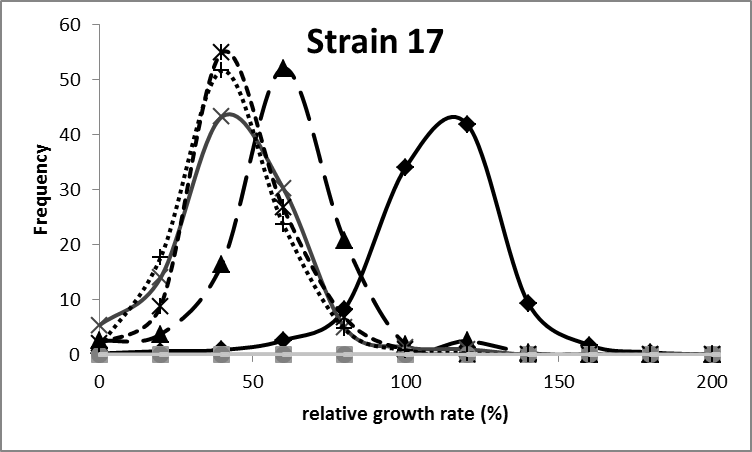


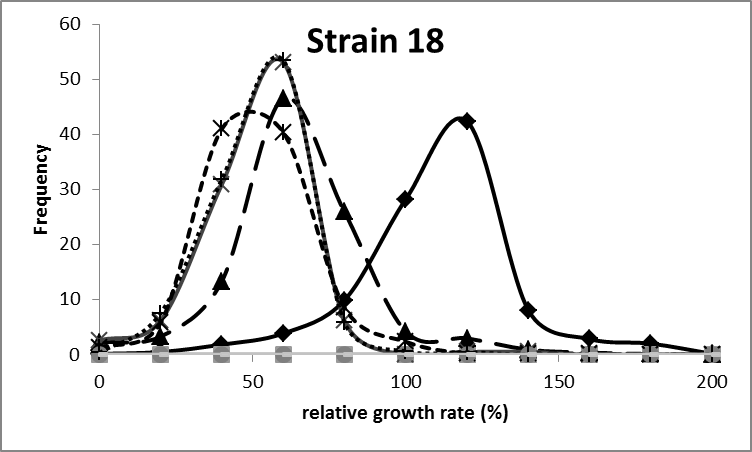


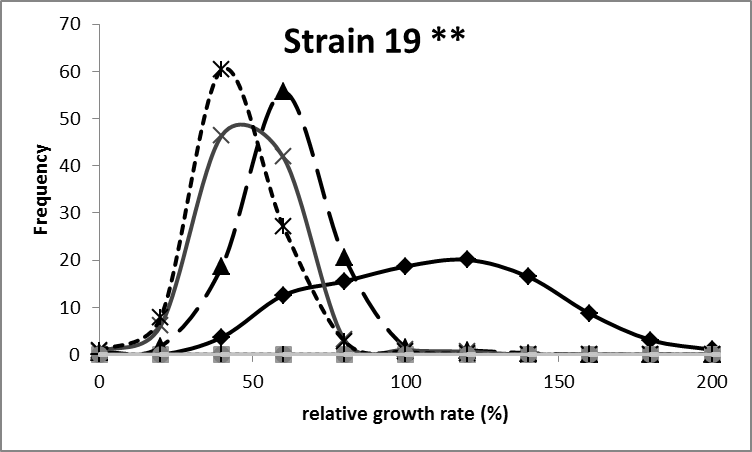


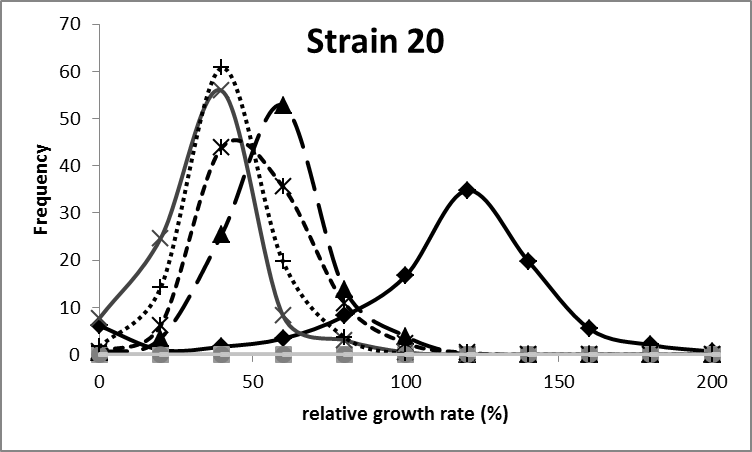


** Relative growth rate as compared to averaged pre-exposure growth rate of the 1, 2 and 4mg/L samples due to contamination in 0 mg/L control


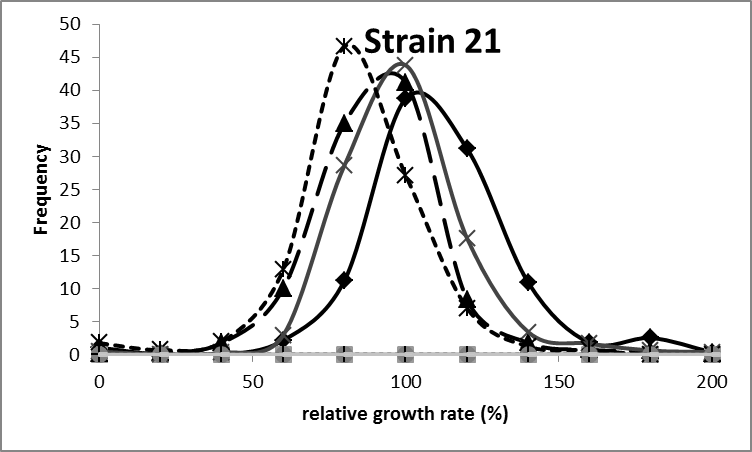


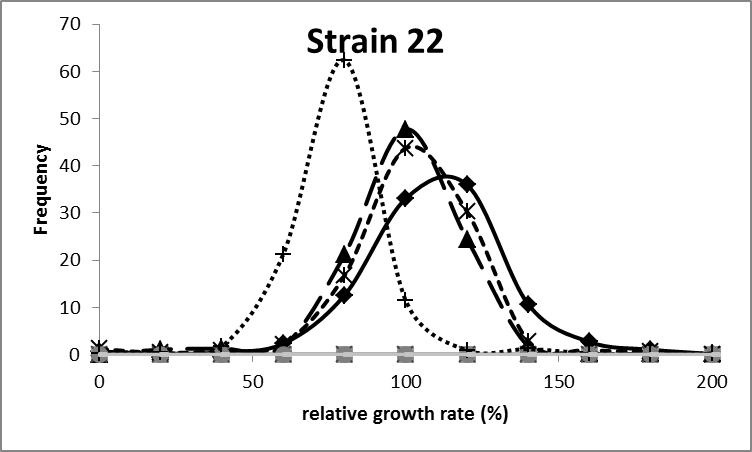


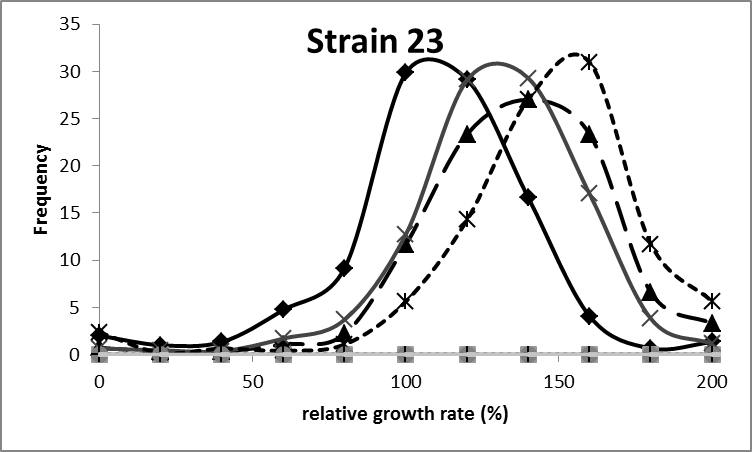


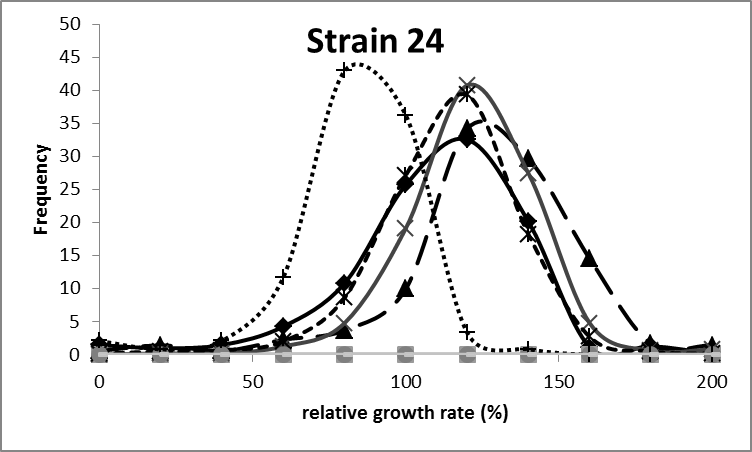

Supplement: Additional file 1 — Frequency distributions of relative growth rates of strains 1–24 between days 7–9. Frequency distributions of the growth rate relative to the averaged unexposed 0 mg/L control are shown for each strain. Distributions are shown with 20% bins, and data are plotted at the upper limit of each bin. (e.g. data over 80-100% is plotted at 100%). Frequency is expressed as the % of total counts. [file 1471-2334-14-380-S1.docx]
